# Help-seeking for mental health problems by employees in the Australian Mining Industry

**DOI:** 10.1186/s12913-016-1755-1

**Published:** 2016-09-21

**Authors:** Ross J. Tynan, Robyn Considine, Jane L. Rich, Jaelea Skehan, John Wiggers, Terry J. Lewin, Carole James, Kerry Inder, Amanda L. Baker, Frances Kay-Lambkin, David Perkins, Brian J. Kelly

**Affiliations:** 1 Hunter Institute of Mental Health, Newcastle, Australia; 2 NHMRC Centre for Research Excellence in Mental Health and Substance Use, University of New South Wales, Old Waratah Post Office, 22 Turton Road, Waratah, NSW Australia; 3 Centre for Resources Health and Safety, University of Newcastle, PO Box 833, Newcastle, 2300 Australia; 4 Centre for Rural and Remote Mental Health, University of Newcastle, HMRI Building, Lot 1 Kookaburra Circuit, New Lambton Heights, NSW 2305 Australia; 5 Population Health, NSW Government Hunter New England Area Health Service, Booth Building, Longworth Avenue, Wallsend, NSW 2287 Australia; 6 School of Medicine and Public Health, University of Newcastle, PO Box 833, Newcastle, 2300 Australia; 7 School of Nursing and Midwifery, University of Newcastle, Newcastle, 2300 Australia; 8 Hunter Building, University Drive, University of Newcastle, Callaghan, NSW 2308 Australia; 9 Richardson Wing, School of Nursing and Midwifery, University Drive, University of Newcastle, Callaghan, Australia

**Keywords:** Mental illness, Help-seeking, Coal mining, Workplace health

## Abstract

**Background:**

The current study examined help-seeking behavior for mental health problems of employees in the mining industry.

**Methods:**

The research involved a paper-based survey completed by a cross-section of employees from eight coalmine sites. The research aimed to investigate the frequency of contact with professional and non-professional sources of support, and to determine the socio-demographic and workplace factors associated.

**Results:**

A total of 1,457 employees participated, of which, 46.6 % of participants reported contact with support to discuss their own mental health within the preceding 12 months. Hierarchical logistic regression revealed a significant contribution of workplace variables, with job security and satisfaction with work significantly associated with help-seeking behavior.

**Conclusions:**

The results provide an insight into the help-seeking behaviour of mining employees, providing useful information to guide mental health workplace program development for the mining industry, and male-dominated industry more broadly.

**Electronic supplementary material:**

The online version of this article (doi:10.1186/s12913-016-1755-1) contains supplementary material, which is available to authorized users.

## Background

Mental illnesses are a leading cause of disability, and consequently represent a substantial economic and societal burden, accounting for 7.4 % of the global burden of disease [1], and 13 % of the national burden of disease in Australia [2]. Treatments for mental illness are effective [3], however, international evidence suggests that help-seeking is low [3–5]. In the 2007 Australian National Survey of Mental Health and Well-being (NSMHWB), only 34.9 % of individuals who met diagnostic criteria for an affective, anxiety, or substance-use disorder reported consulting with a professional service in the preceding 12 months [6]. Efforts to understand why service utilization is low and how to overcome limitations of the current health system to provide effective mental health care are an international imperative [4].

Previous research has demonstrated a number of socio-demographic correlates of professional mental health service use. Internationally, those who use professional services for mental health problems are significantly more likely to be female [4–8], middle-aged [4–6, 8], have a higher level of education [4, 5] and be unmarried [4, 5]. Stigmatizing attitudes, including community, workplace and personal stigma have also previously been associated with a reduction in service uptake [9]. While this evidence is compelling, national population statistics may not necessarily reflect the behaviour of smaller groups, such as workplaces, where little is known about how specific workplace and employment characteristics influence help seeking.

In the Australian coal mining industry, mental health has emerged as a priority for workplace health and safety [10]. The Working Well: Mental Health and Mining study highlighted that the self-reported psychological distress of coal-miners is higher than an employed, age and gender matched sample from the NSMHWB, which may indicate that miners have a greater need for accessing professional mental health services. While little is known about the service use of coalmine employees, there are a number of employment characteristics within the mining industry that may influence help seeking. The Australian mining industry is often characterized as male-dominated, which, in addition to lowering the likelihood of treatment seeking relative to females, may be amplified by a stoic ‘macho culture’, presenting an additional barrier to seeking support [11, 12].

There are a number of additional challenges associated with the mining industry that may impact on service use. Mines are often located in rural or remote regions of Australia, where the availability of professional mental health services is limited [13, 14], with increasing remoteness generally linked with greater challenge in accessing services [15]. Mines that operate under a fly-in fly-out (FIFO) or drive-in drive-out (DIDO) arrangement, whereby staff are required to stay in mine site provided accommodation for the duration of their roster, tend to be even more remote geographically isolated areas. These challenges are compounded by displacement from social networks including both friends and family, long rosters, shift work, and a highly demanding role. Mining employees, thus, may be more susceptible to higher rates of work, family, and health stress [10]. However, the association between the aforementioned characteristics of working in the industry and contact with both professional and non-professional sources of support is unknown.

Within this context, the current study aimed to investigate if, and from whom, employees of the Australian coal mining industry seek help for their own mental health problems. Factors associated with help-seeking behavior are also examined, including employee socio-demographic characteristics, workplace factors and attitudes, and the perception of stigma. Finally, the study set out to assess the relationship between predicted need for professional services and actual professional service utilization.

## Methods

This research was approved by the University of Newcastle Human Research Ethics Committee [H-2013-0135].

### Sample

This cross-sectional study used a quota sampling method with the aim of recruiting a representative sample of employees across the Australian coalmining industry. The approach adopted a number of stratification variables to ensure coverage of coal mining locations (New South Wales [NSW] and Queensland [QLD]; the two primary coal producing states in Australia), mine type (underground or open-cut), and the two primary types of employee commute arrangements (daily commute or long distance commute [DIDO/FIFO]).

### Mine recruitment

Mining companies were approached to provide consent at the company level, which primarily occurred through contact with occupational health and safety managers, with all companies contacted consenting to participate. After company consent, the research team liaised with managers from individual mine sites, and provided an overview of the project to mine site management and occupational health and safety staff, and requested consent at the mine management level. Once consent was received from management, the research team met with key delegates from each mine site to organize the logistics of data collection.

### Participant recruitment and data collection

The data collection protocol was designed to minimize disruption to production, and to accommodate unique logistical considerations of each site. Where possible, the data collection occurred as a component of the sites’ routine training days, which are rostered days assigned for professional development. At sites where training days were unavailable or infrequent, data collection occurred while participants were on shift or during their daily pre-shift meetings. All accessible staff onsite during the specific site visit for data collection were invited to participate.

Two weeks prior to data collection, each participating site was sent a set of study information materials (e.g. PowerPoint slides, posters/flyers) to be displayed in employee common areas to promote awareness of the project. The data collection process involved a research team member visiting the site, providing a brief presentation that gave an overview of the research, as well as a written information statement outlining the research purpose. This material stated that the research was voluntary, confidential, and that participants were free to withdraw at any time. Participation involved completing a brief paper-based survey that took an average of 15 min to complete; return of a completed survey was considered implied consent. Participants were asked to provide a self-generated code (consisting of initials and day/month of birth), to allow potential cross-linkage with future surveys. While it was not anticipated that the content of the survey would cause distress, all participants were provided with a pocket size information card that contained the contact details of a number of different free-to-access support services.

The survey was conducted between December 2013 and March 2015.

### Measures

The survey instrument used for data collection is provided in Additional file 1.

### Outcome variables

#### Help-seeking: The type and frequency of self-reported professional and non-professional contacts for mental health problems

Service utilization and the type of help received were measured using items from the NSMHWB [16, 17]. Participants were given a list of eight professional (e.g. general practitioner, psychologist) and three non-professional (e.g. friend or family member) sources of support, and asked: “In the past 12 months, how many times have you consulted with the following support people to discuss your own mental health problems? *(Note: this can include stress, anxiety, depression or worries about alcohol or drugs)*”. Frequency of contact within the preceding 12 months was measured on a 6-point scale including: 0 times; 1–2 times; 3–5 times; 6–10 times; 11–15 times; and 16+ times; which were assigned the weights of 0, 1.5, 4, 8, 13, and 18 contacts respectively. Participants who reported any contact within the past 12 months were asked to indicate the specific type of help they sought from a list of items covering six domains, including: (1) information; (2) medication; (3) counseling; (4) social intervention; (5) skills training; and/or (6) other.

### Factors associated with contacting professional and non-professional sources of support

To determine factors associated with help seeking, a series of conceptually related variables were grouped into three separate categories (socio-demographic; predicted need for mental health services; and workplace factors and attitudes). This categorization reflected a theoretical interest in determining the independent effect of workplace factors and attitudes on the likelihood of contacting professional or non-professional sources of support, after controlling for the participant’s socio-demographic characteristics and predicted need for professional mental health services.

### Socio-demographic characteristics

Socio-demographic information collected included the participant’s age, gender, relationship/marital status, highest level of education and whether the participant had any dependent children. The number of, and frequency of contact with social connections, and formal and informal group membership was also determined using the Social Network Index [18], with participants scored on a 4-point scale (low, medium, medium-high, high).

### Predicted need for mental health services

To derive an estimate of the need for professional mental health services, the current study used an adapted form of the Predicted Service Need Index (PSNI) [7, 19]. The PSNI takes into account a combination of the participant’s current symptomatology, potential effects from chronic health conditions, and current unhealthy behaviors, which may all reflect an increased need for professional mental health services. Specifically, the PSNI is an aggregate score of participants responses across five health status measures, including: the Kessler-10 (K-10) [20]: a measure of current psychological distress; their self-perceived overall rating of physical and mental health: both assessed using a 5-point Likert scale ranging from ‘poor’ to ‘excellent’; the Alcohol Use Disorders Identification Test (AUDIT) [21]: a measure of problematic alcohol use; and their current smoking status: a single item that measured whether the participant currently smoked cigarettes daily. Each participant received a PSNI score by adding previously determined integer weights (see Perkins et al. [19]) for each of the five health status measures, giving a possible PSNI score range from 0 to 9, with higher scores indicating an increased predicted need for professional mental health services. In the current study, PSNI scores were used both as a dimensional measure (i.e., simple index of increasing predicted need) and a categorical measure, using three previously described [7, 19] categories of need for professional services: low (0–1); medium (2–5); and high (6–9).

### Workplace factors and attitudes

The majority of questions regarding workplace factors were measured using single-item questions including: years working in the mining industry, mine type, commute type, employment category, employee type, shift type, and shift length. The proportion of time at work was also measured based on the participant’s most typical roster, and was calculated by dividing the number of consecutive days at work by the sum of the consecutive days at home plus the number of consecutive days at work.

The survey also included a series of questions to measure participants’ attitudes towards working in the mining industry. Preliminary principal components analyses were conducted to guide the derivation of subscale scores for the following measures of participants’ attitudes:

- *Satisfaction with work:* An average of the responses given to seven items scored on a 5-point scale ranging from ‘very dissatisfied’ to ‘very satisfied’. Items include satisfaction with: Your usual take home pay; Your work prospects; The people you work with; Physical work conditions; The way your section is run; The way your abilities are used; and The interest and skill involved in your job.
- *Work in mining because I love the work, and the roster suits my family*: Average response to two items scored on a 5-point scale ranging from 1: ‘strongly disagree’ to 5: ‘strongly agree’. Items include: I work in coal because I love the work; the roster schedule suits me and my family.
- *Perception of mine’s commitment to mental health*: Average response to five items scored on a 5-point scale ranging from 1: ‘strongly disagree’ to 5: ‘strongly agree’. Items include: This mine would be flexible in offering work adjustments to someone with a mental health problem; This mine provides education and training to supervisors and managers about mental health; The managers at this mine have a good understanding of mental health issues; The mine provides education to employees about mental health; Our workplace policies support the mental health of mine employees.

Workplace attitudes were also assessed using two single-item questions that measured the concern participants had about losing their job and the perception of stigma in the workplace. Stigma was measured by participants self-reporting whether they felt an employee experiencing mental illness would be ‘treated poorly in the workplace if people found out about it’. Perceived stigma was measured on a five-point scale ranging from ‘strongly disagree’ to ‘strongly agree’, with responses categorized as: low stigma (strongly disagree or disagree); unsure (unsure); or high stigma (agree or strongly agree).

### Data analysis

Data were analysed using conventional statistical packages, Microsoft Excel (Version 14) and the Statistical Package for Social Sciences (IBM SPSS version 22; Armonk, NY, USA). Descriptive analysis was used to characterise the socio-demographic and health status of participants. The initial analysis also included a description of contact patterns (both professional and non-professional) associated with discussions about participants’ own mental health within the last 12 months, including the proportion of people who contacted each source of support and the frequency of contact. To facilitate discussion, the types of contact are ranked in order of mean PSNI scores.

The derivation of subscale scores for several of the workplace attitude questions (i.e. satisfaction with work; perceived commitment of mine to the mental health of employees; and the primary reasons for working in mining) was guided by preliminary principal components analyses, based on sets of questions contributing to factors with an eigenvalue above 1.00.

To explore the factors associated with professional and non-professional contacts within the preceding 12 months, we used two hierarchical logistic regression models, with a predetermined order of entry for all associative variables. To examine the unadjusted contributions, in model one, all socio-demographic variables were simultaneously entered at step one, followed by each of the workplace and employment characteristics, which were added separately at step two. For model two, all socio-demographic variables were simultaneously entered at step one, followed by the PSNI category at step 2, and all workplace and employment characteristics added simultaneously at step 3. For all analyses, we report Adjusted Odds Ratios (AOR) with 99 % confidence intervals (CI).

As a partial control for the number of statistical tests, the threshold for statistical significance was set at *p* < 0.01 for all analyses.

## Results

### Participating mines

Eight of the ten mine sites approached agreed to participate in the study. The remaining two mines could not allocate sufficient time for data collection within the data collection period. The eight participating sites contained a representative cross-section of the Australian coal mining industry, with four mining companies, and inclusion of sites from both NSW (five sites) and QLD (three sites), a combination of open cut (three sites) and underground (five sites) mining, as well as mines that operated primarily with daily commute (five sites) and long distance commute (three sites) workforces.

### Sample characteristics

Overall, 1,457 employees across the eight sites completed the survey. Five of the eight sites participated as part of their scheduled training days. Across these five sites, 929 of the 982 employees who attended training days agreed to participate, and returned a completed survey (Average response rate: 95 %, range 92–98 %). At the sites where data collection on training days was not possible, participants were approached at pre-shift meetings. In the case of the latter, an estimate of the participation rate was determined by calculating the number of surveys completed by the number of current mine employees. Across these three sites 2,386 staff were currently employed, and 528 completed the survey (Average participation rate 22 %, range 18–30 %).

In the current analysis, 58 participants were excluded due to incomplete PSNI scores. Sample characteristics of the 1,399 participants who completed all components of the PSNI are shown in Table 1. Consistent with the industry and the employee profile of the participating mines, the sample was primarily young to middle-aged men. Most of the participants were currently married or in a de facto relationship (79.1 %), with dependent children (59.2 %), and reported a trade or apprenticeship as their highest level of education (36.2 %). The sample contained even representation of mine type, and the type and length of shift.

**Table 1:** Sample characteristics of the Working Well: Mental Health and Mining Study baseline survey participants (*n =* 1,399)^a^

Participant Characteristics	*n* (%)	Workplace Characteristics	*n* (%)
*Sex*		*Mine type*	
Male	1215 (86.8)	Open cut	739 (52.8)
Female	175 (12.5)	Underground	660 (47.2)
*Age*		*Commute type*	
< 24	111 (7.9)	FIFO/DIDO	397 (28.4)
25–34	434 (31.0)	Local	999 (71.6)
35–44	428 (30.6)	*Shift type*	
45–54	319 (22.8)	A regular shift	669 (47.9)
55+	97 (6.9)	A rotating shift	702 (50.3)
*Relationship Status*		Other	26 (1.9)
Not Married or de facto	198 (14.2)	*Shift length*	
Married and/or de facto	1106 (79.1)	8 h or less	183 (13.1)
Separated/Divorced/Widowed	85 (6.1)	9–12 h	731 (52.4)
*Dependent Children*		More than 12 h	481 (34.5)
No	571 (40.8)	*Employment Category*	
Yes	828 (59.2)	Managers	67 (4.8)
*Education*		Professional	189 (13.5)
Year 10 or less	279 (19.9)	Trades worker	482 (34.5)
Year 12	170 (12.2)	Machinery Operator	576 (41.2)
Trade	507 (36.2)	Administration or Other	85 (6.1)
Cert/diploma	237 (16.9)	*Years working in Mining*	
Uni/higher degree	199 (14.2)	2 years or less	256 (18.4)
		3 to 10 years	606 (43.3)
		More than 10 years	530 (37.9)

^a^58 participants were excluded with incomplete Predicted Service Need Index (PSNI) scores. However, excluded participants did not significantly differ from respondents who completed all components of the PSNI on any of the tabled variables

### The predicted service need index (PSNI)

The overall PSNI profile was similar to that reported previously in a rural and remote sample [7, 19], with 58.3 % in the low, 32.2 % in the medium and 9.1 % in the high predicted need for professional mental health services categories (see Table 2).

**Table 2:** Health status measures used to calculate the Predicted Service Need Index (PSNI) (*n =* 1,399)

Current Health Status	Weight	*n* (%)	PSNI Mean (SD)
*Overall mental health*			
Good to excellent	0	1143 (81.7)	1.04 (1.01)
Poor/fair	3	256 (18.3)	5.77 (1.39)
*Overall physical health*			
Good to excellent	0	1099 (78.6)	1.32 (1.63)
Poor/fair	1	300 (21.4)	4.07 (2.34)
*Kessler (K-10) score*			
10–15	0	851 (60.8)	0.84 (1.08)
16–24	1	446 (31.9)	2.90 (1.79)
25–50	3	102 (7.3)	6.54 (1.73)
*AUDIT score*			
0–7	0	811 (58.0)	1.23 (1.82)
8+	1	588 (42.0)	2.85 (2.15)
*Current smoker*			
No	0	1136 (81.2)	1.63 (2.02)
Yes	1	263 (18.8)	3.08 (2.16)
*PSNI*			
Low (0–1)		815 (58.3)	
Medium (2–5)		451 (32.2)	
High (5+)		133 (9.1)	
*(Mean = 1.91; SD = 2.13)*			

### Professional and non-professional contacts for mental health problems

Overall, 46.6 % of participants reported that they made contact with at least one professional or non-professional source of support to discuss their own mental health within the preceding 12 months (see Table 3). Non-professional contacts were most common (41.0 %) and of these, friends and/or family members were most commonly identified as the source of support (40.3 %). Friends and family were also contacted more frequently than all other non-professional sources. In terms of professional support, 23.2 % reported contact within the preceding 12 months, with the general practitioner (GP) the most common professional service contacted (18.8 %). Specialist mental health services were associated with the highest mean PSNI scores, whereas the GP and non-professional sources of support were associated with the lowest.

**Table 3:** Professional and non-professional contacts for mental health problems in the preceding 12 months (*n =* 1,399)

Contact type	Any contacts n (%)	Number of times contacted (*for service users*) Mean (SD)	PSNI (*for service users*) Mean (SD)
*Professional contacts:*			
Drug and alcohol counselor	10 (0.7)	5.15 (5.83)	4.80 (2.66)
Psychologist	91 (6.5)	3.64 (2.94)	4.14 (2.66)
Mental health nurse	15 (1.1)	3.67 (4.88)	3.87 (2.83)
Psychiatrist	39 (2.8)	3.46 (3.70)	3.46 (2.49)
Social worker	71 (5.1)	3.00 (2.69)	3.20 (2.67)
General Practitioner	263 (18.8)	2.48 (2.00)	3.05 (2.60)
Specialist doctor or surgeon	45 (3.2)	2.23 (1.58)	2.56 (2.45)
Chemist	61 (4.4)	2.48 (1.89)	2.28 (2.16)
*Total Professional*	325 (23.2)	5.20 (5.94)	2.94 (2.60)
*Non-professional contacts:*			
Clergy	18 (1.3)	3.67 (4.60)	3.44 (2.73)
Complementary therapist	57 (4.1)	2.08 (1.44)	2.65 (2.50)
Friend or family	561 (40.3)	4.47 (5.13)	2.44 (2.34)
*Total non-professional*	573 (41.0)	4.70 (5.32)	2.43 (2.34)
*Any contact (professional or non-professional)*	652 (46.6)	6.72 (7.65)	2.43 (2.35)

**Fig. 1:**
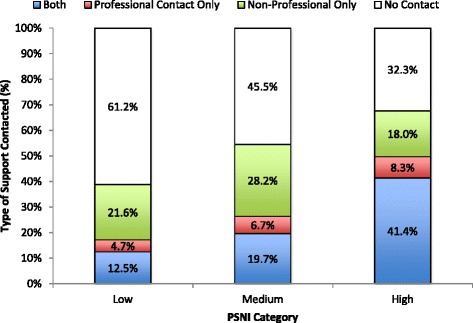
The relationship between PSNI and the type of support service contacted within the preceding 12 months

Figure 1 shows the relationship between the PSNI and contact with both professional and non-professional sources of support within the preceding 12 months, demonstrating that as levels of predicted service need increased, participants were more likely to contact both professional and non-professional sources of support. The collective proportion of participants reporting professional contacts increased as a function of increasing level of PSNI, with 17.2 % in low, 26.4 % in medium, and 49.7 % in the high PSNI stratum reporting contact with a professional service in the preceding 12 months. Of note is that 50.3 % of people in the high PSNI category for professional services had not contacted a professional support service in the preceding 12 months.

For those who reported professional service use within the preceding 12 months, the most common type of help received was medication (45.1 %), followed by counseling (44.2 %) and information about mental illness and its treatments (33.0 %). Fewer participants reported receiving skills training (27.1 %), social intervention (10.0 %) or ‘other’ types of help (24.8 %).

### Factors associated with professional and non-professional help seeking for mental health problems

The relationship between socio-demographics and self-reported contacts to discuss mental health related problems in the preceding 12 months (see Table 4) differed for professional and non-professional sources of support. For non-professional supports, there was a significant effect of age, with younger participants (<21 years) twice as likely to report contact with at least one non-professional source of support, when compared to the two oldest age categories. Gender was significantly associated with contacting both professional and non-professional sources of support, with female participants more than three times as likely to report at least one professional contact and more than twice as likely to have contacted non-professional sources in the preceding 12 months. For professional services, the likelihood of self-reported use decreased with higher levels of education, with those who reported a trade qualification as their highest level of education significantly less likely to report usage than those who reported year 10 or less. Those who were divorced, separated or widowed were significantly more likely to report professional services use than those who were currently married or in a de facto relationship.

**Table 4:** Relationship between socio-demographic characteristics and self-reported contact with professional and non-professional sources of support to discuss own mental health within the last 12 months (*n =* 1,319)

Socio-demographic characteristic	Subgroup *N* (%)	Professional service contact		Non-professional contact	
		*N* (%)	AOR (99 % CI)	*N* (%)	AOR (99 % CI)
Age (years)					
< 24	102 (7.7)	23 (22.5)		54 (52.9)	
25–34	413 (31.3)	94 (22.8)	1.22 (0.57, 2.63)	200 (48.4)	0.83 (0.44, 1.58)
35–44	407 (30.9)	100 (24.6)	1.35 (0.60, 3.02)	166 (40.8)	0.66 (0.33, 1.30)
45–54	298 (22.6)	68 (22.8)	1.05 (0.46, 2.41)	95 (31.9)	0.40 (0.20, 0.82)**
55+	92 (7.0)	19 (20.7)	1.03 (0.37, 2.83)	27 (29.3)	0.39 (0.16, 0.93)*
Gender					
Male	1144 (86.7)	228 (19.9)		435 (38.0)	
Female	168 (12.7)	75 (44.6)	3.25 (1.98, 5.36)**	106 (63.1)	2.48 (1.53, 4.02)**
Education					
Year 10 or less	252 (19.1)	74 (29.4)		90 (35.7)	
Year 12	158 (12.0)	39 (24.7)	0.79 (0.42, 1.49)	52 (32.9)	0.66 (0.37, 1.19)
Trade	480 (36.4)	85 (17.7)	0.59 (0.36, 0.98)*	190 (39.6)	1.13 (0.73, 1.75)
Cert/diploma	227 (17.2)	58 (25.6)	0.75 (0.42, 1.31)	106 (46.7)	1.41 (0.85, 2.34)
Uni/higher degree	196 (14.9)	50 (25.5)	0.68 (0.37, 1.26)	104 (53.1)	1.45 (0.85, 2.50)
Marital Status					
Married/de facto	1043 (79.1)	225 (21.6)		407 (39.0)	
Single	189 (14.3)	45 (23.8)	0.97 (0.53, 1.77)	95 (50.3)	1.07 (0.63, 1.82)
Divorced/separated/widowed	78 (5.9)	34 (43.6)	2.32 (1.17, 4.60)**	40 (51.3)	1.73 (0.89, 3.34)
Dependent Children					
No	540 (40.9)	128 (23.7)		244 (45.2)	
Yes	779 (59.1)	180 (23.1)	1.16 (0.75, 1.78)	301 (38.6)	1.01 (0.70, 1.75)
Social Network Index					
Low	155 (11.8)	45 (29.0)		72 (46.5)	
Medium	512 (38.8)	130 (25.4)	1.04 (0.59, 1.83)	227 (44.3)	1.02 (0.62, 1.68)
Medium-High	474 (35.9)	99 (20.9)	0.84 (0.46, 1.54)	190 (40.1)	0.95 (0.56, 1.62)
High	154 (11.7)	24 (15.6)	0.54 (0.24, 1.21)	46 (29.9)	0.57 (0.34, 1.10)

*Note:* Based on a series of hierarchical logistic regressions, in which all socio-demographic variables were entered simultaneously at step 1 (Table 4), followed by PSNI at step 2 (Table 5) and workplace and employment factors at step 3 (Additional file 2: Table S6): * *p* < 0.01; **p < 0.001. Only participants with a complete set of socio-demographic and workplace characteristics were included. *AOR* adjusted odds ratio, *CI* confidence interval

After accounting for socio-demographic characteristics, there was a significant positive association between PSNI and both professional and non-professional contacts, as shown in Table 5. Participants in the high PSNI category were almost five times more likely to have made contact with at least one professional source of support within the preceding 12 months, and were three times more likely to report contact with at least one non-professional source of support, when compared to those in the lowest PSNI category.

**Table 5:** Aggregate current health service needs and reported professional and non-professional contacts for mental health problems (*n =* 1,319)

PSNI	Sub-group *n*	Professional service contact		Non-professional contact	
		N (%)	AOR (99 % CI)	N (%)	AOR (99 % CI)
Low (0–1)	766 (58.1)	129 (16.8)		264 (34.5)	
Medium (2–5)	425 (32.2)	117 (27.5)	2.01 (1.35, 2.99)**	206 (48.5)	1.85 (1.33, 2.58)**
High (>5)	128 (9.7)	62 (48.4)	4.75 (2.73, 8.25)**	75 (58.6)	3.00 (1.76, 5.10)**

*Note:* Based on a series of hierarchical logistic regressions, in which all socio-demographic variables were entered simultaneously at step 1 (Table 4), followed by PSNI at step 2 (Table 5) and workplace and employment factors at step 3 (Additional file 2: Table S6): * *p* < 0.01; **p < 0.001. Only participants with a complete set of socio-demographic and workplace characteristics were included. *AOR* adjusted odds ratio, *CI* confidence interval

As shown in Table S6 (see Additional file 2), there was a significant association between job security and help seeking, with those who were concerned about losing their job significantly more likely to report contact with both professional and non-professional sources of support within the preceding 12 months. Table S6 (Additional file 2) also shows that satisfaction with work was significantly associated with contacting professional, but not non-professional sources of support, with participants significantly less likely to report professional service use if they were satisfied with work.

### Recruitment method

Differences between recruitment methods were investigated using the same hierarchical regression model approach, with the exception of type of mining and commute type, which were moved to the first level given significant differences in the composition of the workforce for both open cut and underground mines. After controlling for type of mine and commute type, differences observed according to recruitment method included participants from the pre-shift sub-sample more likely to: work on a rotating shift; and be more concerned about losing their job.

## Discussion

This study is the first to assess the impact of working in the Australian coal mining industry on help-seeking behaviour for mental health related problems. Encouragingly, the data indicated that many employees self-reported discussing their own mental health with a variety of professional and non-professional sources of support in the preceding 12 months. When examining the predicted need for professional service use, there was a clear dose–response effect, with those who scored high on the predicted service need index significantly more likely to report contact with both professional and non-professional support. The results provide an insight into the help-seeking behaviour of Australian coal miners, providing useful information to guide mental health program development for the Australian mining industry, and male-dominated industry more broadly.

Despite having a predominantly male sample, the overall proportion of participants who reported professional service use was almost double the national estimate from the NSMHWB (11.9 %) [6], and also higher than other international population based estimates in developed countries which have ranged from 5.6 % in Japan to 17.9 % in the USA [4]. Interestingly, the data were closely aligned to that found in research investigating the professional service use in rural and remote regions of Australia (17 %) [19], and another male-dominated industry in the Australian Defence Force (17.9 %) [22]. It is unclear why service utilization was higher than reported previously. One possibility is that the high proportion of service use reflected a greater need for accessing services, which is supported by the levels of psychological distress observed in the current study being significantly higher than that observed in the NSMHWB [23].

Consistent with much of the population-based research, the GP was the most common source of support contacted, highlighting the importance of primary health care for the treatment of mental health problems. Primary health care is the foundation for high-quality mental health care, with the GP having the potential to identify and treat mental health problems, and refer to specialist mental health services for ongoing treatment when necessary [24]. Interestingly, while the proportion of participants who reported accessing treatment from the GP were more than double the proportion reported in the Australian community previously [6], the proportion of people who contacted more specialist mental health services (e.g. psychiatrist, psychologist) was similar. This highlights the importance of primary health care, particularly in rural and remote regions where mines are typically located, and may reflect the limited availability of more specialist services in these areas.

While the high proportion of employees reporting professional service contact is encouraging, approximately 50 % of those who had cumulative PSNI scores indicative of high level service need had not consulted any professional service in the preceding 12 months. Of note, 18 % of these participants reported contact with non-professional sources of support exclusively. It is possible that this reflects a preference for self-management strategies for mental health problems rather than formal service use, which previous research has shown is common [8], particularly in a “stoic” male-dominated group. It is also important to consider that the prevalence of mental disorders may not be a reliable indicator of perceived service need [25], however, this data would seem to suggest that a considerable proportion of employees who received scores placing them in the high PSNI category may get some benefit from accessing professional treatment.

Almost twice the number of participants indicated that they had made contact with non-professional sources of support in the preceding 12 months, when compared to professional services, with friends and family members the most commonly reported source of contact. This result indicates that a significant proportion sought the help of non-professional contacts exclusively, which may indicate further support for the preference for self-management strategies, in favour of professional service use. It also provides some useful insight into coal miners’ help-seeking behaviour, which may provide some useful guidance for the development of strategies to improve the mental health of coal mine employees. The clear preference for non-professional sources may indicate that providing training to friends and family members on how to appropriately discuss mental health issues, with specific training on how to identify mental health problems, and how to connect people with support when necessary may make these conversations more effective.

### Factors associated with contacting professional and non-professional sources of support

#### Socio-demographic factors

Most of the socio-demographic correlates of help seeking were consistent with those reported in population-based studies [4–6], with female participants and those who were divorced, separated or widowed significantly more likely to report professional service use. Education was significantly associated with professional service use, but in the opposite direction to reported previously [4, 5], with trade qualified employees significantly less likely to report professional service use than those with year 10 education or less. Interestingly, there was a trend towards increased non-professional service use within this subgroup, albeit a non-significant trend, which may further suggest a preference for self-management strategies within this subgroup.

#### Predicted service need

Consistent with previous reports [6, 7, 19], this study found a significant dose–response relationship between predicted need for professional services and actual professional service use, indicating that those identified as high need were significantly more likely to report professional service use in the preceding 12 months.

#### Workplace factors and attitudes

After controlling for socio-demographics and predicted need for services, workplace factors such as the type of mine, commute type, or roster patterns, were not significantly associated with help seeking. Greater concerns about job security and dissatisfaction with the workplace were associated with an increased likelihood of accessing professional services, and may suggest that they acted as additional stressors. It is important to consider the results within the current context of the Australian mining industry, as the study was conducted in a time of significant economic constraints, with many mines reviewing operations, and enforcing involuntary redundancies. While this context needs to be accounted for in interpreting the results, it also provides greater impetus to address the stressors and resultant mental health problems within the industry.

One of the other primary attitudes assessed was the perception of stigma in the workplace. Stigma is often cited as one of the major barriers to accessing treatment for mental health problems, based on the assumption that fear of stigma may inhibit individuals with mental health problems from seeking help [26–28]. However, in population-based studies, the influence of stigma has been mixed, with some reporting that the perception of stigma reduces help seeking [26, 29, 30], whereas others have found no effect [27, 28, 31]. In the current study, we found an unadjusted relationship between the perception of stigma in the workplace and contact with a professional service (model 1, Additional file 2: Table S6); however, this effect disappeared in the multivariate analysis (model 2). Contrary to expectations, these results suggest that the perception of stigma did not have a significant impact on help-seeking behaviour. One caveat to this finding is that the majority of those who reported professional service use had contacted a GP, which may not necessarily carry the stigma of more specialized mental services [31].

#### Limitations

Firstly, the current research uses a cross-sectional design, which does not allow causal inferences to be made.

The data analysed in this study reflects self-report data collected from Australian coal miners. While it is not anticipated that the results were influenced by any form of bias, the data does rely on participants’ accurate recall of professional and non-professional contacts within the preceding 12 months. Given the high proportion of participants who disclosed contact, considerably higher than reported previously, it is unlikely that issues regarding recall influenced the results.

Another important consideration involved differences in recruitment method between sites. The study used two primary data collection methods, with data collected either (1) during site training days; or (2) during pre-shift meetings. As reported, there was considerable variation in the response rate by recruitment method. Further analysis showed that while most factors were not significantly associated with recruitment method, there was a significant association between recruitment method and (1) work schedule and (2) concern about losing job. As concern about losing job was also associated with help seeking, it is unknown to what extent recruitment method affected this association. However, there were no statistically significant differences in the proportion of participants who reported seeking professional or non-professional help between the two recruitment methods. As further evidence to support of the representativeness of the sample, we found a strong correlation between the age, gender and employment category profile for each of the mines that participated in the study with state based employment data.

Another important consideration is the commute distance to the mines. The study did not find any significant association between those who worked in FIFO or DIDO operations compared to those who are employed locally. One caveat to this finding is that the long distance commute mines involved in the current study were primarily DIDO operations, with most of the participants reporting a commute time of less than three hours. Therefore, the data may not necessarily reflect those who work in FIFO operations, or mines that involve a longer distance commute. Importantly, those that are FIFO or have longer distance commute times are potentially more remote, geographically isolated sites, and therefore have greater limitations on the availability of services. Future research is warranted to determine the extent to which longer distance commute times influence help seeking behaviour.

## Conclusions

With a predominantly male workforce, the mining sector may provide unique opportunities for workplace mental health promotion to increase awareness and overcome barriers to help seeking [10]. Workplaces offer a unique setting to deliver mental health promotion and mental illness prevention interventions because they can potentially reach many adults, and incorporate or modify systems and social networks that may enhance or damage a person’s mental health [32, 33]. Understanding the personal (socio-demographic), structural (such as rosters or site location) or attitudinal barriers (such as mental illness stigma) that facilitate or inhibit a person from seeking support for mental health problems is important to guide the development of workplace mental health programs. Understanding the contributions of different barriers to mental health service use is vital to ensure that efforts to optimise service use are appropriately directed.

The findings of this research provide useful insights into the help-seeking behaviour of Australian coal miners, and male-dominated industry more broadly. The results show that a considerable proportion of coal miners are actively engaged in activities to improve their mental health, with high self-reported use of both professional and non-professional sources of support. The strong preference for non-professional sources of support observed in the current study is a key finding that may suggest that development of mental health programs providing education to friends and family members may be of some benefit.

## Additional files

Additional file 1: Survey. (DOC 377 kb) Additional file 2: Table S6. Workplace factors and attitudes associated with professional and non-professional help seeking (*n =* 1,319). (DOC 97 kb)

## Abbreviations

AOR: Adjusted odds ratio
AUDIT: Alcohol use disorder identification test
DIDO: Drive-in drive-out
FIFO: Fly-in fly-out
GP: General practitioner
NSMHWB: National survey of mental health and well-being
NSW: New South Wales
QLD: Queensland
