# Supplementary material for: Help-seeking for mental health problems by employees in the Australian Mining Industry
Source: BMC Health Serv Res. 2016 Sep 21;16:498. doi: 10.1186/s12913-016-1755-1 (PMC5031264; doi:10.1186/s12913-016-1755-1)
Supplement: Additional file 1: — Survey. (DOC 377 kb) [file 12913_2016_1755_MOESM1_ESM.doc]

**Additional File 1 - Survey**

**
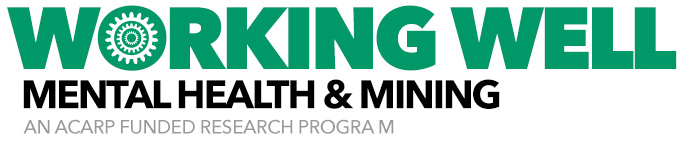
**

**
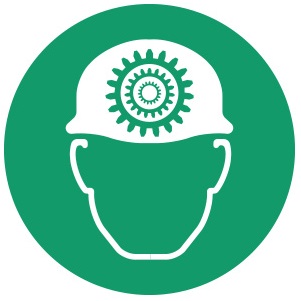
**

**Consent**

After reading the information sheet, if you agree to participate in this survey, please give your consent by ticking the ‘I agree’ below.

**I agree to participate**  🞎

Please place the initials of your first and last name (2 letters) and the numbers of the day and month of your birthday (4 numbers).

|  | **Example**  Tom Brown is born on 7^th^ October 1961. His code is TB0710 | | |
| --- | --- | --- | --- |
| Your initials and date of birth | |  |  |

**Tips for Completing the Survey**

For the purpose of this research, it is important that you answer all questions honestly, or to the best of your knowledge. Your answers will remain strictly confidential, and will only be used for research purposes. The information you provide will not be personally identifiable, and will only appear as grouped data for all employees at the mine.

If you need any assistance completing the survey, speak to one of the research project team in the room.

**The first group of questions ask about your age, family and other personal characteristics.**

1. In which age group (in years) do you belong?

| 🞎 | 17-24 | 🞎 | 25-34 | 🞎 | 35-44 | 🞎 | 45-54 | 🞎 | 55-64 | 🞎 | 65-74 | 🞎 | 75+ |
| --- | --- | --- | --- | --- | --- | --- | --- | --- | --- | --- | --- | --- | --- |

| 🞎 | Male | 🞎 | Female |
| --- | --- | --- | --- |

1. **What is your gender?**
2. Do you identify as being of Aboriginal and/or Torres Strait Islander Origin?

| 🞎 | No | 🞎 | Aboriginal | 🞎 | Torres Strait Islander | 🞎 | Both Aboriginal and Torres Strait Islander |
| --- | --- | --- | --- | --- | --- | --- | --- |

1. What best describes your marital or relationship status? (tick all that apply)

| 🞎 | Single (Never married) | 🞎 | In a relationship (Not living together) | 🞎 | Separated (but not divorced) | 🞎 | Widowed |
| --- | --- | --- | --- | --- | --- | --- | --- |
| 🞎 | Married | 🞎 | In a relationship (Living together) | 🞎 | Divorced |  |  |

1. **How many dependent children (children aged under 15, or students aged 15-24) do you have?**
   _____________Children.
2. Which of the following best describes your household?

| 🞎 | Couple family with children | 🞎 | Multiple family household | 🞎 | One parent family with children | 🞎 | Group household |
| --- | --- | --- | --- | --- | --- | --- | --- |
| 🞎 | Couple only | 🞎 | Lone person | 🞎 | Other one family household |  |  |

1. In which country were you/ born?

| 🞎 | Australia | 🞎 | New Zealand | 🞎 | Vietnam | 🞎 | Greece |
| --- | --- | --- | --- | --- | --- | --- | --- |
| 🞎 | England | 🞎 | Italy | 🞎 | Scotland | 🞎 | Germany |
| 🞎 | Philippines | 🞎 | India | 🞎 | Netherlands | 🞎 | Other |

1. What is the highest level of education you have obtained?

| 🞎 | No school qualification or other qualifications | 🞎 | School or intermediate certificate (Year 10 or equivalent) | 🞎 | Higher school or leaving certificate (Year 12 or equivalent). |
| --- | --- | --- | --- | --- | --- |
| 🞎 | Trade /apprenticeship (e.g. Electrician) | 🞎 | Certificate/ diploma (e.g. child care, technician) | 🞎 | University or higher degree |

**The next group of questions are about your current work situation**

1. How would you best describe your employment category?

| 🞎 | Manager | 🞎 | Technicians and Trades Worker | 🞎 | Labourer | 🞎 | Other |
| --- | --- | --- | --- | --- | --- | --- | --- |
| 🞎 | Professional (e.g. engineer, OH&S professional) | 🞎 | Clerical and Administrative Worker | 🞎 | Machinery Operators and Driver |  |  |

| 🞎 | Employed full time | 🞎 | Employed part time |
| --- | --- | --- | --- |

1. **What is your employment status?**
2. What is the status of your employment contract?

| 🞎 | Employed on a permanent or ongoing basis | 🞎 | Employed on a casual basis | 🞎 | Other |
| --- | --- | --- | --- | --- | --- |
| 🞎 | Employed on a fixed-term contract | 🞎 | Contractor/Sub contractor |  |  |

1. Which of these best describes your current work schedule at this mine?

| 🞎 | A regular shift (day, afternoon or evening) | 🞎 | Panel roster | 🞎 | On call | 🞎 | Other |
| --- | --- | --- | --- | --- | --- | --- | --- |
| 🞎 | A rotating shift (e.g. changes from days to afternoons to nights) | 🞎 | Split shift (two distinct periods each day) | 🞎 | Irregular schedule |  |  |

1. What is the length of hours for your most common shift or time at work?

| 🞎 | Less than 8 hours | 🞎 | 8 hours | 🞎 | 9-11 hours |
| --- | --- | --- | --- | --- | --- |
| 🞎 | 12 hours | 🞎 | More than 12 hours |  |  |

1. Would you consider yourself as a fly-in, fly-out (FIFO) or drive-in, drive-out (DIDO) worker? (where your mine is a long way from your home base meaning you have to live away from home while at work)

| 🞎 | Yes | 🞎 | No |
| --- | --- | --- | --- |

1. **How many hours does it take for you to get from your home to your place of work?**

______________Hours

1. **On your most typical roster, how many consecutive days are you at work?**

______________Days

1. **On your most typical roster, how many consecutive days are you at home or away from work?**

______________Days

1. How much control do you have over…

|  | **None** | **Not Very Much** | **A fair amount** | **Quite a lot** | **Complete Control** |
| --- | --- | --- | --- | --- | --- |
| The specific shifts that you work? |  |  |  |  |  |
| The specific start and finish times of the shifts you work? |  |  |  |  |  |

1. **How many years have you been working in the mining industry?** ________________ years
2. **How long have you been working at this mine?**  ________________ years

**The next series of questions ask about how you have been feeling in the last four weeks**

1. In the last 4 weeks about often did you feel…

|  | **None of the time** | **A little of the time** | **Some of the time** | **Most of the time** | **All of the time** |
| --- | --- | --- | --- | --- | --- |
| Tired out for no good reason? |  |  |  |  |  |
| Nervous? |  |  |  |  |  |
| So nervous that nothing could calm you down? |  |  |  |  |  |
| Hopeless? |  |  |  |  |  |
| Restless or fidgety |  |  |  |  |  |
| So restless you could not sit still? |  |  |  |  |  |
| Depressed? |  |  |  |  |  |
| That everything was an effort? |  |  |  |  |  |
| So sad that nothing could cheer you up? |  |  |  |  |  |
| Worthless? |  |  |  |  |  |

1. **In the last four weeks, how many days were you totally unable to work, study or manage your day-to-day activities because of these feelings**?

____________number of days

1. **Aside from [that day-those days], in the last four weeks, how many days were you able to work, study or manage your day-to-day activities, but had to cut down on what you did because of these feelings?**

____________number of days

1. **In the last four weeks, how many times have you seen a doctor or other health professional about these feelings?**

____________number of consultations

1. In the last 4 weeks, how often have physical health problems been the main cause of these feelings?

| 🞎 | None of the time | 🞎 | A little of the time | 🞎 | Some of the time | 🞎 | Most of the time | 🞎 | All of the time |
| --- | --- | --- | --- | --- | --- | --- | --- | --- | --- |

**The next series of questions ask about how your health and wellbeing.**

1. During the last four weeks, how would you rate your:

|  | **Poor** | **Fair** | **Good** | **Very Good** | **Excellent** |
| --- | --- | --- | --- | --- | --- |
| Overall physical health? |  |  |  |  |  |
| Overall mental health? |  |  |  |  |  |
| Overall relationships with your family, close friends and acquaintances? |  |  |  |  |  |
| Ability to perform everyday duties and tasks (e.g. at home, at work, studying, shopping, looking after yourself)? |  |  |  |  |  |

1. **During the last four weeks, how would you rate your:**

|  | **Not at all satisfied** | **Somewhat satisfied** | **Moderately satisfied** | **Very satisfied** | **Extremely satisfied** |
| --- | --- | --- | --- | --- | --- |
| Overall satisfaction with the community in which you live? |  |  |  |  |  |
| Overall satisfaction with life? |  |  |  |  |  |

1. How would you rate your stress from the events that have occurred in your life (e.g., stressful events in your family, at work or in your community?

| 🞎 | Not at all stressed | 🞎 | Somewhat stressed | 🞎 | Moderately stressed | 🞎 | Very stressed | 🞎 | Extremely stressed |
| --- | --- | --- | --- | --- | --- | --- | --- | --- | --- |

1. Has a doctor EVER told you that you have any of the following: (Please tick all that apply)

| 🞎 | Heart attack or angina | 🞎 | High blood pressure | 🞎 | Diabetes | 🞎 | Depression |
| --- | --- | --- | --- | --- | --- | --- | --- |
| 🞎 | Other heart disease | 🞎 | Stroke | 🞎 | Migraine | 🞎 | Anxiety |
| 🞎 | High cholesterol | 🞎 | Cancer | 🞎 | Obesity | 🞎 | Drug or alcohol problems |

1. Which of the following categories best describes your smoking status?

| 🞎 | Never smoked | 🞎 | Current smoker, less often than daily |
| --- | --- | --- | --- |
| 🞎 | Ex-smoker | 🞎 | Current daily smoker |

**The following questions are about your attitudes towards people with a mental illness.**

1. People who experience a mental illness would be:

|  | **Strongly disagree** | **Disagree** | **Unsure** | **Agree** | **Strongly Agree** |
| --- | --- | --- | --- | --- | --- |
| Treated differently by their friends if they found out about their illness. |  |  |  |  |  |
| Treated differently by their colleagues if they found out about their illness |  |  |  |  |  |
| Treated poorly in this workplace if people found out about it. |  |  |  |  |  |

**The next series of questions relate to a case study about a miner named Tom.**

#### Please read the following story and answer the questions that relate to Tom's situation.

####

Tom is 35 and has been working in this mine for about six years. He is married with two children. In the last few months he has lost weight, and has been feeling unusually sad and miserable. He appears more irritable than normal and has occasionally commented about problems at home. He seems to find it difficult to concentrate at work and appears to be tired and forgetful. He is becoming more distant from colleagues who he usually gets along well with. He has more days off than usual and mentions that he has been hung-over and unable to do some of the family activities.

1. What do you think best describes the problem (if any) with Tom? (tick one)

| 🞎 | Physical Health problem | 🞎 | Depression | 🞎 | Anxiety | 🞎 | Schizophrenia |
| --- | --- | --- | --- | --- | --- | --- | --- |
| 🞎 | Substance Abuse Problem | 🞎 | Alcohol Problem | 🞎 | There appears to be nothing wrong with him |  |  |

1. Do you think if Tom sought help from the following sources it would be helpful, harmful or neither helpful nor harmful?

| **Source of help** | **Harmful** | **Neither** | **Helpful** |
| --- | --- | --- | --- |
| Talk to someone trustworthy |  |  |  |
| Talk to family/friends |  |  |  |
| Talk to supervisor |  |  |  |
| See a GP |  |  |  |
| See a psychiatrist |  |  |  |
| See a counsellor or psychologist |  |  |  |
| Contact telephone helpline |  |  |  |
| Seek no help from these sources |  |  |  |

1. **Do you think that the following treatments would be helpful, harmful or neither helpful nor harmful to Tom?**

| **Treatment** | **Harmful** | **Neither** | **Helpful** |
| --- | --- | --- | --- |
| Vitamins, minerals, herbal medicines |  |  |  |
| Pain relievers (e.g. aspirin, panadol) |  |  |  |
| Anti-depressants |  |  |  |
| Antibiotics |  |  |  |
| Sleeping pills |  |  |  |
| Tranquilizers (e.g. valium) |  |  |  |
| Alcohol |  |  |  |

**The following questions are about your attitudes towards suicide**

1. **Using the scale below, please rate how much you agree with the descriptions of people who take their own lives (suicide). In general, people who suicide are…**

|  | **Strongly Disagree** | **Disagree** | **Unsure** | **Agree** | **Strongly Agree** |
| --- | --- | --- | --- | --- | --- |
| Shallow |  |  |  |  |  |
| Lonely |  |  |  |  |  |
| Pathetic |  |  |  |  |  |
| Strong |  |  |  |  |  |
| Immoral |  |  |  |  |  |
| Isolated |  |  |  |  |  |
| Stupid |  |  |  |  |  |
| Noble |  |  |  |  |  |
| Irresponsible |  |  |  |  |  |
| Lost |  |  |  |  |  |
| An embarrassment |  |  |  |  |  |
| Dedicated |  |  |  |  |  |
| Cowardly |  |  |  |  |  |
| Disconnected |  |  |  |  |  |
| Vengeful |  |  |  |  |  |
| Brave |  |  |  |  |  |

**The next group of questions are about help seeking behaviour.**

1. **In the past 12 months, how many times have you consulted with the following support people to discuss your own mental health problems? (*Note: this can include stress, anxiety, depression or worries about alcohol or drugs***)

|  | **0 times** | **1-2 times** | **3-5 times** | **6-10 times** | **11-15 times** | **16+** |
| --- | --- | --- | --- | --- | --- | --- |
| General Practitioner |  |  |  |  |  |  |
| Psychiatrist |  |  |  |  |  |  |
| Psychologist |  |  |  |  |  |  |
| Drug and alcohol counsellor |  |  |  |  |  |  |
| Mental health nurse |  |  |  |  |  |  |
| Social worker, counsellor, or occupational therapist |  |  |  |  |  |  |
| Chemist for professional advice |  |  |  |  |  |  |
| Specialist doctor or surgeon including cardiologist, gynaecologist (women), or urologist (males) |  |  |  |  |  |  |
| Complementary/alternative therapist such as herbalist or naturopath |  |  |  |  |  |  |
| Friend or family member |  |  |  |  |  |  |
| Clergy |  |  |  |  |  |  |

1. **From the people contacted above, which of the following forms of help did you receive? (*tick all that apply*)**

| 🞎 | Information about mental illness, its treatments, and available services | 🞎 | Medicine or tablets |
| --- | --- | --- | --- |
| 🞎 | Counselling-help to talk through your problems | 🞎 | Help to sort out housing or money problems |
| 🞎 | Help to improve your ability to work, or to use your time in other ways | 🞎 | Help to improve your ability to look after yourself or your home |
| 🞎 | Help to meet people for support and company | 🞎 | Other |

**The next group of questions are about your work experiences in this mine.**

1. **About how many hours altogether did you work in the past 7 days?** (*If more than 97, enter 97*)

_________________Number of hours (00-97)

1. **How many hours does your employer expect you to work in a typical 7-day week?** (*If it varies, estimate the average. If more than 97, enter 97*)

_________________Number of hours (00-97)

1. **In the past 4 weeks** (28 days), how many days did you…

|  | **Number of Days** |
| --- | --- |
| Miss an **entire** work day because of problems with your physical or mental health? |  |
| Miss **part** of a work day because of problems with your physical or mental health? |  |
| Come in early, go home late, or work on your day off? |  |

1. **On a scale from 0 to 10, where 0 is the worst job performance anyone could have at your job and 10 is the performance of a top worker, how would you rate:**

The usual performance of **most** workers in a job similar to yours? (please circle)

| Worst Performance | | | | | Top Performance | | | | | |
| --- | --- | --- | --- | --- | --- | --- | --- | --- | --- | --- |
| 0 | 1 | 2 | 3 | 4 | 5 | 6 | 7 | 8 | 9 | 10 |

Your **usual** job performance over the **past year or two**?

| Worst Performance | | | | | Top Performance | | | | | |
| --- | --- | --- | --- | --- | --- | --- | --- | --- | --- | --- |
| 0 | 1 | 2 | 3 | 4 | 5 | 6 | 7 | 8 | 9 | 10 |

Your **overall** job performance on the days you worked during the **past 4 weeks** (28 days)?

| Worst Performance | | | | | Top Performance | | | | | |
| --- | --- | --- | --- | --- | --- | --- | --- | --- | --- | --- |
| 0 | 1 | 2 | 3 | 4 | 5 | 6 | 7 | 8 | 9 | 10 |

1. In the past 4 weeks (28 days), did you have any of the following experiences at work?

|  | **Yes** | **No** |
| --- | --- | --- |
| Any special work success or achievement? |  |  |
| Any special work failure? |  |  |
| An accident that caused either damage, work delay, a near miss, or safety risk? |  |  |

1. **About how many days of work did you miss in the past 12 months because of a work related accident, injury, or poisoning?**

_________________days

1. Indicate how often the following things occur:

|  | **Often** | **Sometimes** | **Seldom** | **Never/ Almost Never** | **Not applicable** |
| --- | --- | --- | --- | --- | --- |
| Do you have to work very fast? |  |  |  |  |  |
| Do you have to work very intensively? |  |  |  |  |  |
| Do you have enough time to do everything? |  |  |  |  |  |
| Are your tasks such that others can help if you do not have enough time? |  |  |  |  |  |
| Do you have the possibility of learning new things through work? |  |  |  |  |  |
| Does your work demand a high level of skill or expertise? |  |  |  |  |  |
| Does your job require you to take the initiative? |  |  |  |  |  |
| Do you have to do the same thing over and over again? |  |  |  |  |  |
| Do you have a choice in deciding how you work? |  |  |  |  |  |
| Do you have a choice in deciding what you do at work? |  |  |  |  |  |
| Does your job provide you with a variety of interesting things to do? |  |  |  |  |  |
| Is your immediate superior willing to listen to your problems |  |  |  |  |  |

1. How satisfied are you with the following?

|  | **Very Satisfied** | **Satisfied** | **Unsure** | **Dissatisfied** | **Very dissatisfied** |
| --- | --- | --- | --- | --- | --- |
| Your usual take home pay |  |  |  |  |  |
| Your work prospects |  |  |  |  |  |
| The people you work with |  |  |  |  |  |
| Physical working conditions |  |  |  |  |  |
| The way your section is run |  |  |  |  |  |
| The way your abilities are used |  |  |  |  |  |
| The interest and skill involved in your job |  |  |  |  |  |

1. **Are you worried about losing your job?**

| 🞎 | Not at all | 🞎 | Mildly worried | 🞎 | Moderately worried | 🞎 | Very worried | 🞎 | Extremely worried |
| --- | --- | --- | --- | --- | --- | --- | --- | --- | --- |

1. The following questions are about your feelings related to your income and work.

|  | **Strongly disagree** | **Disagree** | **Unsure** | **Agree** | **Strongly Agree** |
| --- | --- | --- | --- | --- | --- |
| I work in coal mining because I love the work |  |  |  |  |  |
| The pay is the main reason I work in coal mining |  |  |  |  |  |
| The roster schedule suits me and my family |  |  |  |  |  |
| I have financial commitments that mean I have to continue to work in coal mining because of the salary levels |  |  |  |  |  |
| I would prefer to work in another job but can’t afford to leave because of my financial commitments |  |  |  |  |  |

**The next group of questions are about your social and community relationships**

1. How many…

|  | **None** | **1-2** | **3-5** | **6-9** | **10 or more** |
| --- | --- | --- | --- | --- | --- |
| Close friends do you have? (People that you feel at ease with, can talk to about private matters, and can call on for help). |  |  |  |  |  |
| Work colleagues do you have that you feel close to? |  |  |  |  |  |
| Relatives do you have that you feel close to? |  |  |  |  |  |
| Of these friends or relatives do you see at least once a month? |  |  |  |  |  |

1. Do you belong to any of these kinds of groups?

|  | **No** | **Yes** |
| --- | --- | --- |
| A social or recreational group |  |  |
| A labour union, commercial group, professional organisation |  |  |
| Church group |  |  |
| A group concerned with children e.g. boy scouts, parents and friends etc. |  |  |
| A group concerned with community betterment, charity, or service? |  |  |
| Any other group |  |  |

**The following questions are about the sort of things your current mine does or might do for mental health**

Can you indicate your level of agreement with the following statements regarding your mine’s approach to mental health in the workplace?

|  | **Strongly Disagree** | **Disagree** | **Unsure** | **Agree** | **Strongly Agree** |
| --- | --- | --- | --- | --- | --- |
| This mine would be flexible in offering work adjustments to someone with a mental health problem |  |  |  |  |  |
| This mine provides education and training to supervisors and managers about mental health |  |  |  |  |  |
| The managers in the mine have a good understanding of mental health issues |  |  |  |  |  |
| This mine provides education to employees about mental health |  |  |  |  |  |
| Our workplace policies support the mental health of mine employees |  |  |  |  |  |

**The next group of questions are about your alcohol consumption and how this affects you and others around you**

*Please note, the information collected will be used for research purposes only. Any information you provide will remain strictly confidential, and no personally identifying information linking you to your response will be disclosed to your employer.*

1. How often do you have a drink containing alcohol?

| 🞎 | Never | 🞎 | Monthly or less | 🞎 | 2-4 times a month | 🞎 | 2-3 times a week | 🞎 | 4 or more times a week |
| --- | --- | --- | --- | --- | --- | --- | --- | --- | --- |

1. How many drinks containing alcohol do you have on a typical day when you are drinking?

| 🞎 | 1 or 2 | 🞎 | 3 or 4 | 🞎 | 5 or 6 | 🞎 | 7 to 9 | 🞎 | 10 |
| --- | --- | --- | --- | --- | --- | --- | --- | --- | --- |

1. How often do you have six or more drinks on one occasion?

| 🞎 | Never | 🞎 | Less than monthly | 🞎 | Monthly | 🞎 | Weekly | 🞎 | Daily or almost daily |
| --- | --- | --- | --- | --- | --- | --- | --- | --- | --- |

1. How often during the last year have you found that you were not able to stop drinking once you had started?

| 🞎 | Never | 🞎 | Less than monthly | 🞎 | Monthly | 🞎 | Weekly | 🞎 | Daily or almost daily |
| --- | --- | --- | --- | --- | --- | --- | --- | --- | --- |

1. How often during the last year have you failed to do what was normally expected of you because of drinking?

| 🞎 | Never | 🞎 | Less than monthly | 🞎 | Monthly | 🞎 | Weekly | 🞎 | Daily or almost daily |
| --- | --- | --- | --- | --- | --- | --- | --- | --- | --- |

1. How often during the last year have you needed a first drink in the morning to get yourself going after a heavy drinking session?

| 🞎 | Never | 🞎 | Less than monthly | 🞎 | Monthly | 🞎 | Weekly | 🞎 | Daily or almost daily |
| --- | --- | --- | --- | --- | --- | --- | --- | --- | --- |

1. How often during the last year have you had a feeling of guilt or remorse after drinking?

| 🞎 | Never | 🞎 | Less than monthly | 🞎 | Monthly | 🞎 | Weekly | 🞎 | Daily or almost daily |
| --- | --- | --- | --- | --- | --- | --- | --- | --- | --- |

1. How often during the last year have you been unable to remember what happened the night before because of your drinking?

| 🞎 | Never | 🞎 | Less than monthly | 🞎 | Monthly | 🞎 | Weekly | 🞎 | Daily or almost daily |
| --- | --- | --- | --- | --- | --- | --- | --- | --- | --- |

1. Have you or someone else been injured because of your drinking?

| 🞎 | No | 🞎 | Yes, but not in the last year | 🞎 | Yes, during the last year |
| --- | --- | --- | --- | --- | --- |

1. Has a relative, friend, doctor, or other health care worker been concerned about your drinking or suggested you cut down?

| 🞎 | No | 🞎 | Yes, but not in the last year | 🞎 | Yes, during the last year |
| --- | --- | --- | --- | --- | --- |

**The next three questions are about any illicit drugs you may have used in the last month.**

*A reminder, all information provided is strictly confidential, and no personally identifying data will be disclosed to your employer.*

1. Have you used marijuana/cannabis in the last month?

| 🞎 | No, have never tried it | 🞎 | No, not in the last month | 🞎 | Yes, once or twice | 🞎 | Yes, about once a week | 🞎 | Yes, daily |
| --- | --- | --- | --- | --- | --- | --- | --- | --- | --- |

1. Have you used any synthetic drugs (e.g. KRONIK, synthetic marijuana) in the last month?

| 🞎 | No, have never tried it | 🞎 | No, not in the last month | 🞎 | Yes, once or twice | 🞎 | Yes, about once a week | 🞎 | Yes, daily |
| --- | --- | --- | --- | --- | --- | --- | --- | --- | --- |

1. Have you used other illicit drugs (e.g. ecstasy, amphetamines, cocaine) in the last month?

| 🞎 | No, have never tried it | 🞎 | No, not in the last month | 🞎 | Yes, once or twice | 🞎 | Yes, about once a week | 🞎 | Yes, daily |
| --- | --- | --- | --- | --- | --- | --- | --- | --- | --- |

**Thank you for your participation in this research project. Rest assured that any information you have provided will remain strictly confidential.**

You can keep looking after your mental health by:

Staying active

Eating and sleeping well

Staying connected with family and friends

Only drinking in moderation

Please take a card from the researcher for services of who you can contact to learn more about mental health.

***Please return the paper to the researcher.***
