# Supplementary material for: Help-seeking for mental health problems by employees in the Australian Mining Industry
Source: BMC Health Serv Res. 2016 Sep 21;16:498. doi: 10.1186/s12913-016-1755-1 (PMC5031264; doi:10.1186/s12913-016-1755-1)
Supplement: Additional file 2: Table S6. — Workplace factors and attitudes associated with professional and non-professional help seeking (n = 1,319). (DOC 97 kb) [file 12913_2016_1755_MOESM2_ESM.doc]

Additional File 2: Table S6. Workplace factors and attitudes associated with professional and non-professional help seeking (n=1,319)

|  |  | Professional contact | | | Non-Professional contact | | |
| --- | --- | --- | --- | --- | --- | --- | --- |
|  | Subgroup n | N (%) | Model 1  N (99% CI) | Model 2  N (99% CI) | N (%) | Model 1  N (99% CI) | Model 2  N (99% CI) |
| ***Workplace Factors*** |  |  |  |  |  |  |  |
| Mine type |  |  |  |  |  |  |  |
| Open Cut | 715 (54.2) | 176 (24.6) |  |  | 308 (43.1) |  |  |
| Underground | 604 (45.8) | 132 (21.9) | 1.05 (0.73, 1.51) | 0.88 (0.53, 1.46) | 237 (39.2) | 0.95 (0.70, 1.30) | 0.85 (0.56, 1.28) |
| Commute type |  |  |  |  |  |  |  |
| Daily commute (local) | 939 (71.2) | 230 (24.5) |  |  | 383 (40.8) |  |  |
| Long distance commute (FIFO or DIDO) | 380 (28.8) | 78 (20.5) | 0.82 (0.55, 1.23) | 0.77 (0.48, 1.22) | 162 (42.6) | 1.10 (0.79, 1.54) | 1.10 (0.75, 1.60) |
| Years working in Mining |  |  |  |  |  |  |  |
| 2 years or less | 240 (18.2) | 50 (20.8) |  |  | 106 (44.2) |  |  |
| 3 – 10 years | 586 (44.4) | 144 (24.6) | 1.33 (0.79, 2.24) | 1.20 (0.68, 2.12) | 251 (42.8) | 0.96 (0.62, 1.47) | 0.83 (0.52, 1.32) |
| More than 10 years | 493 (37.4) | 114 (23.1) | 1.44 (0.82, 2.56) | 1.32 (0.69, 2.52) | 188 (38.1) | 1.10 (0.68, 1.76) | 0.90 (0.53, 1.53) |
| Employment Category |  |  |  |  |  |  |  |
| Manager | 67 (5.1) | 14 (20.9) |  |  | 31 (46.3) |  |  |
| Professional | 186 (14.1) | 44 (23.7) | 0.99 (0.38, 2.56) | 1.05 (0.37, 2.93) | 100 (53.8) | 1.02 (0.47, 2.23) | 0.96 (0.42, 2.18) |
| Technician or Trades worker | 462 (35.0) | 83 (18.0) | 1.06 (0.42, 2.73) | 1.06 (0.38, 3.00) | 187 (40.5) | 0.82 (0.38, 1.76) | 0.72 (0.31, 1.64) |
| Machinery operator or labourer | 519 (39.3) | 137 (26.4) | 1.42 (0.57, 3.56) | 1.54 (0.54, 4.37) | 185 (35.6) | 0.71 (0.33, 1.51) | 0.61 (0.26, 1.42) |
| Employee type |  |  |  |  |  |  |  |
| Mine Employee | 1202 (91.1) | 283 (23.5) |  |  | 495 (41.2) |  |  |
| Contractor or subcontractor | 117 (8.9) | 25 (21.4) | 0.72 (0.37, 1.38) | 0.73 (0.35, 1.52) | 50 (42.7) | 0.96 (0.56, 1.64) | 0.92 (0.51, 1.66) |
| Shift type |  |  |  |  |  |  |  |
| Regular | 634 (48.1) | 160 (25.2) |  |  | 282 (44.5) |  |  |
| Rotating | 660 (50.0) | 142 (21.5) | 0.81 (0.56, 1.17) | 0.62 (0.37, 1.04) | 254 (38.5) | 0.86 (0.63, 1.18) | 0.83 (0.54, 1.29) |
| Other | 25 (1.9) | 6 (24.0) |  | 0.93 (0.23, 3.83) | 9 (36.0) | 0.77 (0.25, 2.37) | 0.76 (0.23, 2.54) |
| Shift Length |  |  |  |  |  |  |  |
| 8 hours or less | 164 (12.4) | 50 (30.5) |  |  | 70 (42.7) |  |  |
| 9 – 12 hours | 697 (52.8) | 153 (22.0) | 0.78 (0.46, 1.35) | 0.78 (0.41, 1.50) | 305 (43.8) | 1.10 (0.67, 1.80) | 0.92 (0.51, 1.66) |
| More than 12 hours | 458 (34.7) | 105 (22.9) | 0.77 (0.44, 1.35) | 0.74 (0.34, 1.61) | 170 (37.1) | 0.92 (0.55, 1.53) | 0.70 (0.35, 1.40) |
| Proportion of days at work |  |  |  |  |  |  |  |
| <1:1 | 69 (5.3) | 17 (24.3) |  |  | 24 (34.3) |  |  |
| 1:1 | 584 (44.3) | 124 (21.2) | 1.13 (0.50, 2.54) | 1.34 (0.54, 3.31) | 239 (40.9) | 1.62 (0.79, 3.36) | 1.79 (0.82, 3.91) |
| >1:1, <=2.5:1 | 266 (20.2) | 68 (25.6) | 1.36 (0.58, 3.21) | 1.73 (0.67, 4.49) | 105 (39.5) | 1.37 (0.63, 2.94) | 1.52 (0.66, 3.50) |
| >2.5:1 | 399 (30.3) | 99 (24.8) | 1.13 (0.49, 2.60) | 0.98 (0.35, 2.78) | 177 (44.4) | 1.43 (0.68, 3.00) | 1.17 (0.48, 2.86) |
|  |  |  |  |  |  |  |  |
| ***Attitudes*** |  |  |  |  |  |  |  |
| Satisfaction with work |  |  |  |  |  |  |  |
| Strongly disagree or Disagree | 70 (5.3) | 28 (40.0) |  |  | 36 (51.4) |  |  |
| Unsure | 355 (26.9) | 117 (33.0) | 0.66 (0.32, 1.36) | 0.70 (0.32, 1.56) | 170 (47.9) | 0.75 (0.37, 1.50) | 0.81 (0.38, 1.72) |
| Agree or Strongly Agree | 894 (67.8) | 163 (18.2) | 0.31 (0.15, 0.61)** | 0.37 (0.16, 0.86)* | 339 (37.9) | 0.53 (0.27, 1.03) | 0.63 (0.29, 1.36) |
| Concern about losing job |  |  |  |  |  |  |  |
| Not at all | 318 (24.1) | 49 (15.4) |  |  | 98 (30.8) |  |  |
| Mildly or moderately | 775 (58.8) | 190 (24.5) | 1.72 (1.07, 2.77)* | 1.64 (1.01, 2.76)* | 340 (43.9) | 1.70 (1.16, 2.49)** | 1.68 (1.13, 2.51)** |
| Very or Extremely | 226 (17.1) | 69 (30.5) | 2.20 (1.24, 3.92)** | 1.37 (0.72, 2.61) | 107 (47.3) | 2.14 (1.31, 3.49)** | 1.76 (1.03, 3.01)* |
| The roster schedule suits my family and I love the work |  |  |  |  |  |  |  |
| Strongly disagree or Disagree | 155 (11.8) | 46 (29.7) |  |  | 76 (49.0) |  |  |
| Unsure | 403 (30.6) | 92 (22.8) | 0.71 (0.40, 1.25) | 0.88 (0.47, 1.65) | 160 (39.7) | 0.62 (0.37, 1.03) | 0.64 (0.37, 1.11) |
| Agree or Strongly Agree | 761 (57.7) | 170 (22.3) | 0.66 (0.39, 1.13) | 1.18 (0.63, 2.20) | 309 (40.6) | 0.66 (0.41, 1.06) | 0.75 (0.44, 1.30) |
| Perceive the mine is committed to the mental health of its employees |  |  |  |  |  |  |  |
| Strongly disagree or Disagree | 254 (19.3) | 76 (29.9) |  |  | 116 (45.7) |  |  |
| Unsure | 647 (49.1) | 149 (23.0) | 0.71 (0.45, 1.11) | 1.04 (0.62, 1.74) | 253 (39.1) | 0.71 (0.48, 1.07) | 0.91 (0.58, 1.42) |
| Agree or Strongly Agree | 418 (31.7) | 83 (19.9) | 0.59 (0.35, 0.98)* | 1.07 (0.58, 1.97) | 176 (42.1) | 0.86 (0.55, 1.33) | 1.30 (0.77, 2.17) |
| Administration or other | 85 (6.4) | 30 (35.3) | 1.37 (0.46, 4.01) | 1.31 (0.40, 4.26) | 42 (49.4) | 0.89 (0.35, 2.25) | 0.82 (0.31, 2.19) |
| Mental illness stigma in the workplace |  |  |  |  |  |  |  |
| Low | 658 (49.9) | 128 (19.5) |  |  | 260 (39.5) |  |  |
| Unsure | 387 (29.3) | 95 (24.5) | 2.10 (0.44, 9.95) | 1.16 (0.74, 1.82) | 169 (43.7) | 1.22 (0.85, 1.73) | 1.16 (0.80, 1.68) |
| High | 274 (20.8) | 85 (31.0) | 1.34 (1.07, 1.68)** | 1.18 (0.70, 1.99) | 116 (42.3) | 1.19 (0.80, 1.77) | 0.88 (0.56, 1.40) |

*Note:* Based on a series of hierarchical logistic regressions, in which all socio-demographic variables were entered simultaneously at step 1 (Table 4), followed by PSNI at step 2 (Table 5) and workplace and employment factors at step 3 (Table 6): * *p*<0.01; ***p*<0.001. Only participants with a complete set of socio-demographic and workplace characteristics were included. AOR: Adjusted Odds Ratio; CI: confidence interval.
